# Evaluation of the attractiveness of different gingival zeniths in smile esthetics

**DOI:** 10.1590/2177-6709.23.5.047-057.oar

**Published:** 2018

**Authors:** Suzy Nomura, Karina Maria Salvatore Freitas, Paula Patrícia Cotrin da Silva, Fabricio Pinelli Valarelli, Rodrigo Hermont Cançado, Marcos Roberto de Freitas, Renata Cristina Gobbi de Oliveira, Ricardo Cesar Gobbi de Oliveira

**Affiliations:** 1 Private Practice (São Paulo/SP, Brazil).; 2 Centro Universitário Ingá, Departamento de Ortodontia (Maringá/PR, Brazil).; 3 Universidade de São Paulo, Faculdade de Odontologia de Bauru, Programa de Pós-graduação em Odontologia (Bauru/SP, Brazil).; 4 Universidade de São Paulo, Faculdade de Odontologia de Bauru, Departamento de Ortodontia, Odontopediatria e Saúde Coletiva (Bauru/SP, Brazil).

**Keywords:** Smile, Gingival zenith, Esthetics, Attractiveness

## Abstract

**Objective:**

To evaluate the smile attractiveness of different gingival zeniths by general dentists, orthodontists and laypersons and the esthetic perception in the symmetric and asymmetric changes in gingival zeniths.

**Methods:**

Posed photographs of five patients were taken and digitally manipulated in Keynote software, in the gingival zenith region, in increments of 0.5 to 1mm in maxillary central and lateral incisors, symmetrically and asymmetrically, in nine different ways for each patient. The photos were then uploaded to a website, where evaluators (general dentists, orthodontists and laypersons) could observe and vote according to their esthetic perception, scoring from 1 to 10, 1 being the least attractive and 10 the more attractive. Kruskal-Wallis and Mann-Whitney tests were used for comparison.

**Results:**

Asymmetric gingival zeniths were less attractive than symmetrical gingival zeniths; gingival zenith differences greater than 1mm were perceptible in the smile attractiveness, both by laypersons, general dentists and orthodontists. When comparing maxillary central incisors with maxillary lateral incisors, the aesthetic change performed in the central incisors are more perceptible than those performed in lateral incisors, both symmetrical and asymmetrical. In a general way, orthodontists and general dentists are more critical in the evaluation and perception of gingival zenith changes, with the laypersons perceiving this change only from 1mm of maxillary right central incisor asymmetrical change.

**Conclusions:**

Asymmetric gingival zeniths are less attractive than symmetrical ones. Gingival zenith differences greater than 1mm are perceptible in the smile attractiveness. Orthodontists and general dentists are more critical in evaluating smile esthetics.

## RESUMO

**Objetivo:**

avaliar a atratividade e a percepção estética do sorriso com alterações simétricas e assimétricas dos zênites gengivais, por leigos, dentistas e ortodontistas.

**Métodos:**

foram tiradas fotografias posadas de cinco pacientes, de forma padronizada. Cada fotografia foi digitalmente manipulada no *software* Keynote, na região do zênite gengival, em incrementos de 0,5 a 1 mm, nos incisivos centrais e laterais superiores, de maneira simétrica e assimétrica, de nove formas diferentes para cada paciente. Posteriormente, essas fotografias foram dispostas em um *website* para que os avaliadores pudessem observá-las e, conforme sua percepção estética, dessem notas de 1 a 10, sendo 1 para pouco atrativa e 10 para muito atrativa. Foram utilizados os testes Kruskal-Wallis e Mann-Whitney para as comparações.

**Resultados:**

os zênites gengivais assimétricos foram considerados menos atrativos do que os simétricos. Diferenças entre os zênites gengivais maiores que 1 mm foram perceptíveis na atratividade do sorriso por todos os grupos de avaliadores. Quando comparados os incisivos centrais e laterais superiores, as alterações estéticas feitas nos incisivos centrais foram mais perceptíveis do que as feitas nos laterais, tanto as simétricas quanto as assimétricas. Os ortodontistas e os clínicos gerais foram mais críticos ao avaliar e perceber as alterações, com os leigos percebendo essa mudança somente a partir de 1 mm de alteração assimétrica no incisivo central superior direito.

**Conclusões:**

zênites gengivais assimétricos são menos atrativos do que os simétricos. Diferenças nos zênites maiores que 1 mm são perceptíveis na atratividade do sorriso. Ortodontistas e clínicos gerais são mais críticos ao avaliar sorrisos.

## INTRODUCTION

Dentofacial aesthetics have a great importance in social attractiveness of the individual, and in this context the maxillary incisors play a fundamental role. Patients who have a normal and aligned relationship of the incisors have generally been classified as more friendly, popular, intelligent and with greater chances of getting a job than individuals who present disharmony in these teeth.^1^ ^,2^ The perception of dental aesthetics, however, varies significantly among patients and professionals from different areas, despite substantial efforts to establish common treatment parameters.^3^ Esthetic Dentistry is, more and more, arousing interest and playing an important role in the dentists clinical routine, as well as in patients’ lives, especially nowadays where media promotes the beauty in wonderful faces and perfect smiles, and they are all related with good health and mental/physical well-being.^4^ The integration among various specialties became basic and necessary in today’s dentistry to perform a complete dental treatment.

Some aesthetic parameters for evaluation of the smile aesthetics by laypersons are already established. They are: 0-2mm diastema, 0-3mm midline discrepancy, 5-16mm buccal corridors, 1.5-4mm gingival exposure, 0-4° occlusal canting and 2-5mm overbite.^5^ However, the aesthetic parameters for evaluation of smile attractiveness with different gingival zeniths are not well known, mainly among laypersons. Besides that, in an evaluation of what would be less aesthetic between asymmetries of the incisal edges or asymmetries of the gingival margins, laypersons and dental professionals considered the latter less attractive.^6^

According to Miller^7^ a well-grounded eye is capable to detect something that is unbalanced, out of harmony and out of symmetry.

The gingival zenith is the most apical point of the gingival tissue and is located distal to the longitudinal axis of maxillary centrals and canines. In the maxillary lateral incisors and all mandibular incisors, they should coincide with longitudinal dental axis.^4^ Any change of these positions could generate aesthetic disharmony, and depending on the size of this disharmony, be noticed even by laypersons.

General dentists and orthodontists are more severe when judging their patients’ smiles? The gingival esthetic is as important to laypersons as to dentists and orthodontists? Different gingival zeniths are perceivable for general dentists, orthodontists and laypersons?

In order to answer these questions, the aim of this study was to evaluate the smile attractiveness of different gingival zeniths by general dentists, orthodontists and laypersons, besides to evaluate the esthetic perception of symmetric and asymmetric changes in the gingival zenith and to compare these differences among central and lateral maxillary incisors. 

## MATERIAL AND METHODS

The study has been approved by research ethics committee of *Centro Universitário Ingá* (UNINGÁ) under protocol number 23950913.6.0000.5220. All participants in the study signed a free and informed consent form.

The sample size calculation has been based on an alpha significance level of 5% (0.05) and a beta of 20% (0.20) to achieve a 80% power of the test to detect a minimum difference of 1 (±1.36) for the 0 to 10 scores (using a attractiveness scale in which 0 = hardly attractive; 5 = attractive, and 10 = very attractive).^8^Thus, the sample size calculation showed the need for at least 30 individuals in each group. 

The sample was selected from 5 patients, males and females, with attractive smiles, using the following criteria: complete dentition including second molars, no dental anomalies (shape and number), no anterior diastemas and no active periodontal disease, Class I malocclusion and matching midlines. The smile’s picture was obtained from several photographic records of posed smiles, so the most attractive could be selected.^9^ ^-^ ^11^ The photos were taken by the same operator with a digital Nikon D7000 camera, macrolens 105mm and R1C1 twin flash mounted on a tripod.

The smile pictures were obtained with the patient seated in front of the operator, 60 cm away from the camera lens. The researcher and patient chairs were adjusted to keep the camera lens in the same height of patient lips, adjusting the tripod. Patients were trained to keep the natural head position, in a upright posture, focusing an imaginary point in the same height of their eyes, resulting in a horizontal vision axis.^12^

Patients were instructed to give a pleasant posed smile as natural as possible, with their teeth in the maximum intercuspation position. Several frames were obtained from the same patient to choose a more pleasant picture to be included in the sample. For standardization, all the photographs were obtained in manual mode, colored, with fine quality, ISO 100, diaphragm aperture of 22 and shutter speed of 125.

**Figure 1:**
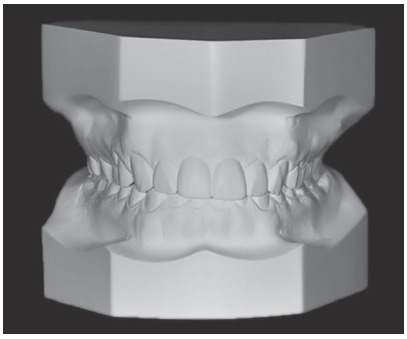
Dental cast.

Each photo was digitally manipulated in Keynote software (Apple, USA) to be evaluated by laypersons, general dentists and orthodontists, then they were uploaded to a specifically designed website, in which evaluators could choose the smile attractiveness in different gingival zenith positions. This digitally manipulation was also made to reduce distracting factors or number of variables, as explained: photos were cropped to correct small head alterations and to diminish the examination area, remaining visible only maxillary and mandibular incisors with their adjacent soft tissue, including lips. Height and width standardization was also made in the selected pictures, to cut them all in the same size proportion. To the edited images, a magnification was made to keep the proportions of teeth and gingivae, using the patient real incisor height measured in the dental casts (Fig 1). This way, all photos could reach a real size proportion of the dental structures and their soft tissue, when seen at the same distance. 

All photos were converted from color to black and white to reduce the confounding factors.^13^

**Figure 2:**
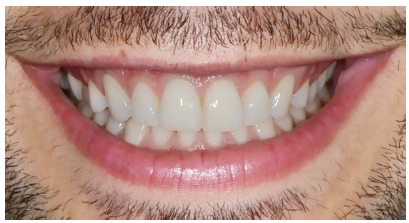
Ideal smile.

**Figure 3:**
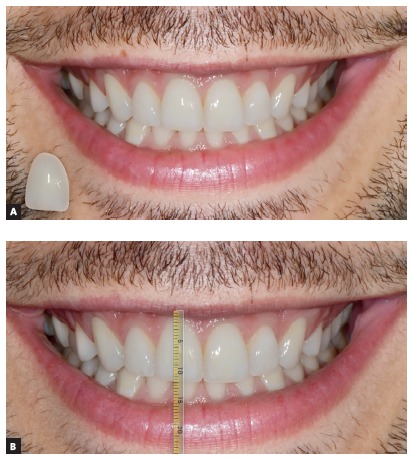
A) Keynote mask; B) Maxillary right central incisor with increase of 1mm performed with Keynote mask.

To the different gingival zenith evaluation, five patients with attractive smile and well balanced facial proportions were selected to use their initial images as ideal (Fig 2). The original photograph (ideal smile) was then manipulated, by using a image processing software (Keynote) with different levels of gingival zeniths alterations (Fig 3).

The gingival zenith alterations were:

1) Ideal: ideal zenith, adjustments with increments smaller than 0.5mm in the initial photos, approaching to the smile considered ideal.
2) Right maxillary lateral incisor (U2R): asymmetrically altered zenith with an 0.5-mm increase in the right maxillary lateral incisor.
3) Maxillary lateral incisors (U2) ½ = symmetrically altered zenith with 0.5-mm increase in maxillary lateral incisors.
4) Right maxillary central incisor (U1R) ½ = asymmetrically altered zenith with 0.5-mm addition in the upper right central incisor.
5) Maxillary central incisors (U1) ½ = symmetrically altered zenith with 0.5-mm enlargement in the upper central incisors.
6) Right maxillary lateral incisor (U2R) 1 = asymmetrically altered zenith with 1-mm increase in right maxillary lateral incisor.
7) Maxillary lateral incisors (U2) 1 = symmetrically altered zenith with 1-mm increase in maxillary lateral incisors.
8) Right maxillary central incisor (U2R) 1 = asymmetrically altered zenith with 1-mm increase in right upper central incisor.
9) Maxillary central incisors (U1) 1 = symmetrically altered zenith with 1-mm increase in maxillary central incisors.

**Figure 4:**
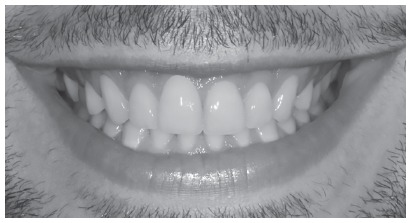
Final manipulated black and white photo.

After photographs digital manipulation, a print screen was taken, then the image was converted to black and white. The black and white space was defined as standard, using the same percentages to the following settings: saturation, brightness, sharpness and contrast, for each subject group (Fig 4).

Three groups of raters were used in this study: laypeople, general dentists and orthodontists. In this research, layperson was defined as a subject with no formal education in dentistry or dental hygiene, however, but studying or already finished high school, with minimal age of 17 years and maximum age of 75 years. General dentists should be graduated in Dentistry and could have or not a specialization in any Dentistry area, except Orthodontics. Orthodontists raters were considered as a general dentist that already had finished their specialization, master degree or PhD in Orthodontics.^10^ Each rater received the links to access the internet website where they could access and evaluate the photos. 

Laypersons group comprised 71 subjects with mean age of 30.78 years, general dentists group comprised 30 subjects (20 female and 10 male) with mean age of 38. 4 years; and orthodontists group comprised 56 subjects (22 female and 34 male) with mean age of 38 years.

**Figure 5:**
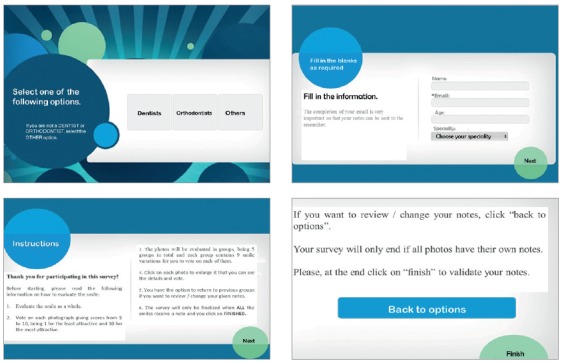
Website print screens.

**Figure 6:**
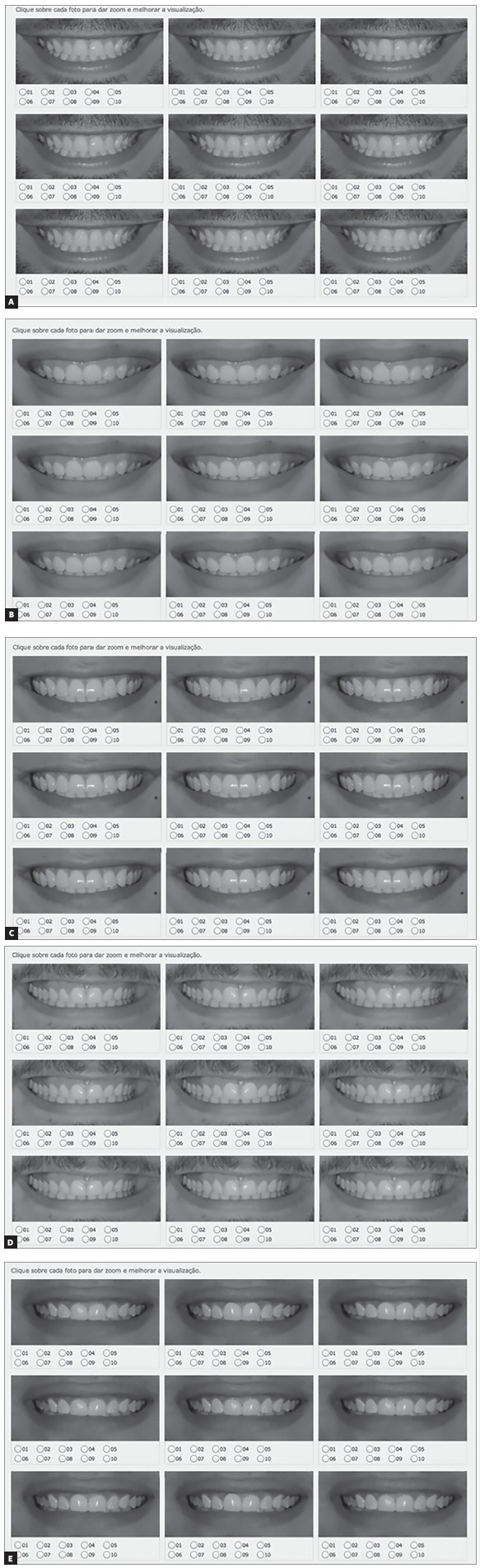
Altered gingival zeniths: A) patient 1, B) patient 2, C) patient 3, D) patient 4, E) patient 5.

The smile attractiveness evaluation was made through website visualization. When accessing the web address *www.digitalorthosmile.com* (Fig 5), the evaluator went through the following phases sequence to perform the analysis of the different gingival zeniths: Free and Informed Consent Form (questionnaire elaborated in a simplified way, specifically for dentists, laypersons and orthodontists); simple instructions to facilitate the access and understanding of the research participants; and the photos gallery called Dental Aesthetic Gallery. Before starting the research, the evaluators visualized all 9 pictures of each patient. Then, they rated their preferences in this group and they went on to the next one until the end of the voting in the 5 patients in the sample (Fig 6). It was stipulated that 5 smiles photographs would be a sufficient number to generate a reliability of results, and even 45 (5 x 9) being a large number, they were evaluated in groups of 9, allowing the evaluators to give the note that they considered more applicable.

The raters could stay as long as necessary to evaluate the images, and could come back in the web site at any time to better judge any smile, with the possibility of enlarging the image for better verification one by one. Once completed and sent the final answers, they could not re-open the questionnaire to change the scores. After finalizing the votes, some orientations were given to the evaluators and the data were collected.

All data were stored in an database accessible via internet only by the researchers.

### Statistical analysis

Intra-examiner error was performed to verify the rater’s response reproducibility. It was calculated using a new survey with new answers by 20 examiners, one month later. Weighted Kappa test was used.^14^ To determine the gender ratio, the three sample groups were compared to each other, using Chi-Square test. To determine the age compatibility between the sample groups, the Variance Analysis (ANOVA) and Tukey’s test were used. The non-parametric Kruskal-Wallis test was used to compare the smile changes among all groups and separately between laypersons, dentists and orthodontists. To compare each smile change between the younger and older groups, Mann-Whitney non-parametric test was used.

## RESULTS

Comparing the ages between laypersons, general dentists and orthodontists (Table 1), results indicated a significant difference between laypersons group and general dentists and orthodontists groups. Laypersons were younger, with mean age of 30.78 years, while general dentists had a mean age of 38.4 years and orthodontists had a mean age of 38 years.

**Table 1:** Age comparison among laypersons, dentists and orthodontists (One-way ANOVA test and Tukey’s test).

Variable	Laypersons	Dentists	Orthodontists	p
	(n=71)	(n=30)	(n=56)	
	Mean (S.D.)	Mean (S.D.)	Mean (S.D.)	
Age (years)	30.78 (12.11)^A^	38.40 (8.75)^B^	38.00 (10.43)^B^	0.000*

*Statistically significant for p<0.05

Smile changes comparison among all groups and separately between each other (Table 2) has been statistically significant, with some relevant results to be considered: to laypersons, the right maxillary central incisor changes were statistically significant; to orthodontists, the 0.5-mm asymmetrical changes in the right maxillary central incisor were statistically significant, however, there were no significant differences in 0.5-mm maxillary central incisors symmetrical changes. In all three groups, right maxillary central incisor had the major significant statistically difference. Among all groups, it was observed that for the 1-mm increase in the gingival margins all they had statistically significant differences. 

**Table 2:** Smile changes comparison among different gingival zeniths for all evaluators and for the 3 groups of evaluators separately (Non-parametric Kruskal-Wallis test).

	IDEAL	U2R ½	U2 ½	U1R ½	U1 ½	U2R 1	U2 1	U1R 1	U1 1	p
	Median (Mean) I.R. (s.d.)	Median (Mean) I.R. (s.d.)	Median (Mean) I.R. (s.d.)	Median (Mean) I.R. (s.d.)	Median (Mean) I.R. (s.d.)	Median (Mean) I.R. (s.d.)	Median (Mean) I.R. (s.d.)	Median (Mean) I.R. (s.d.)	Median (Mean) I.R. (s.d.)	
All (n=785)	7.00 (6.61)	6.00 (6.39)	7.00 (6.48)	6.00 (5.90)	6.00 (6.28)	6.00 (6.11)	6.00 (6.02)	5.00 (4.95)	6.00 (5.89)	0.000*
	1.90(3.00)	1.91 (3.00)	3.00 (1.90)	2.00 (2.00)	3.00 (2.05)	3.00 (2.24)	2.00 (1.96)	2.00 (2.19)	4.00 (2.25)	
	A	AC	A	B	AC	BC	BC	D	B	
Laypersons	7.00 (6.49)	7.00 (6.44)	7.00 (6.46)	6.00 (6.09)	6.00 (6.29)	7.00 (6.60)	6.00 (6.26)	5.00 (5.39)	6.00 (6.25)	0.000*
	3.00 (2.09)	3.00 (2.11)	3.00 (2.07)	3.00 (2.05)	3.00 (2.14)	3.00 (2.03)	3.00 (2.04)	3.00 (2.13)	3.00 (2.28)	
	A	A	A	A	A	A	A	B	A	
Dentists	7.00 (6.60)	6.00 (6.26)	7.00 (6.39)	5.00 (5.52)	6.00 (5.85)	6.00 (5.63)	6.00 (5.78)	4.00 (4.37)	5.00 (4.93)	0.000*
	3.00 (1.60)	2.00 (1.53)	1.00 (1.57)	3.00 (1.81)	2.00 (1.87)	3.00 (1.72)	2.00 (1.70)	3.00 (1.94)	3.00 (2.06)	
	A	ACD	AC	BCDF	BCD	BDF	BCD	E	EF	
Orthodontists	7.00 (6.76)	6.00 (6.40)	7.00 (6.54)	6.00 (5.87)	7.00 (6.50)	6.00 (5.76)	6.00 (5.85)	5.00 (4.71)	6.00 (5.95)	0.000*
	3.00 (1.78)	3.00 (1.83)	3.00 (1.84)	3.00 (2.00)	3.00 (2.00)	3.00 (2.60)	3.00 (1.96)	3.00 (2.27)	4.00 (2.18)	
	A	ABD	A	BC	A	C	CD	E	BC	

*Statistically significant for p<0.05. Different letters in a row indicate the presence of statistically significant difference.

When comparing each smile change between laypersons, general dentists and orthodontists (Table 3), it was statistically significant for the 0.5-mm maxillary right central incisor change. Laypersons and general dentists had a smaller statistical difference in the 0.5mm and 1.00mm maxillary central incisors symmetrical changes.

**Table 3:** Comparison of each gingival zenith change among laypersons, dentists and orthodontists (Non-parametric Kruskal-Wallis test).

ZENITH CHANGES	Laypeople (n=71)	Dentists (n=30)	Orthodontists (n=56)	P
	Median (Mean) I.R. (S.D.)	Median (Mean) I.R. (S.D.)	Median (Mean) I.R. (S.D.)	
IDEAL	7.00 (6.49)	7.00 (6.60)	7.00 (6.76)	0.31
	3.00 (2.09)	3.00 (1.60)	3.00 (1.78)	
U2R ½	7.00 (6.44)	6.00 (6.26)	6.00 (6.40)	0.505
	3.00 (2.11)	2.00 (1.53)	3.00 (1.83)	
U2 ½	7.00 (6.46)	7.00 (6.39)	7.00 (6.54)	0.705
	3.00 (2.07)	1.00 (1.57)	3.00 (1.84)	
U1R ½	6.00 (6.09)	5.00 (5.52)	6.00 (5.87)	0.004*
	3.00 (2.05)	3.00 (1.81)	3.00 (2.00)	
	A	B	AB	
U1 ½	6.00 (6.29)	6.00(5.85)	7.00 (6.50)	0.003*
	3.00 (2.14)	2.00(1.87)	3.00 (2.00)	
	A	B	A	
U2R 1	7.00 (6.60)	6.00 (5.63)	6.00 (5.76)	0.000*
	3.00 (2.03)	3.00 (1.72)	3.00 (2.60)	
	A	B	B	
U2 1	6.00 (6.26)	6.00 (5.78)	6.00 (5.85)	0.008*
	3.00 (2.04)	2.00 (1.70)	3.00 (1.96)	
	A	B	B	
U1R 1	5.00 (5.39)	4.00 (4.37)	5.00 (4.71)	0.000*
	3.00 (2.13)	3.00 (1.94)	3.00 (2.27)	
	A	B	B	
U1 1	6.00 (6.25)	5.00 (4.93)	6.00 (5.95)	0.000*
	3.00 (2.28)	3.00 (2.06)	4.00 (2.18)	
	A	B	A	

*Statistically significant for p<0.05. Different letters in a row indicate the presence of statistically significant difference.

In a comparison performed dividing each group in two parts (Table 4) - a younger part (from 17 to 40 years old) and an older part (from 41 to 75 years old) - smile changes in the right maxillary central incisors, right maxillary lateral incisor, right maxillary central incisor were statically significant, with the older group always giving lower scores than the younger one, and this, as seen before, corroborate the fact that dentists and orthodontists groups had the older evaluators, and the laypersons group had the younger subjects.

**Table 4:** Comparison of each smile change between younger and older groups (Mann-Whitney non-parametric test).

ZENITH CHANGES	Younger (n=530)	Older (n=225)	p
	Median (Mean) I.R. (s.d.)	Median (Mean) I.R. (s.d.)	
IDEAL	7.00 (6.64)	7.00 (6.55)	0.761
	3.00 (1.84)	3.00 (2.02)	
U2R ½	7.00 (6.39)	6.00 (6.39)	0.851
	3.00 (1.90)	3.00 (1.94)	
U2 0.5	7.00 (6.48)	7.00 (6.47)	0.921
	3.00 (1.88)	3.00 (1.96)	
U1R 0.5	6.00 (5.91)	6.00 (5.87)	0.676
	3.00 (1.99)	3.00 (2.02)	
U1 0.5	6.00 (6.41)	7.00 (6.00)	0.003*
	3.00 (2.01)	3.00 (2.12)	
U2R 1	6.00 (6.16)	6.00 (6.02)	0.571
	3.00 (2.30)	3.00 (2.10)	
U2 1	6.00 (6.11)	6.00 (5.83)	0.044*
	3.00 (1.95)	3.00 (1.99)	
U1R 1	5.00 (5.06)	5.00 (4.72)	0.038*
	3.00 (2.15)	3.00 (2.24)	
U1 1	6.00 (6.14)	6.00 (5.36)	0.000*
	3.00 (2.19)	4.00 (2.30)	

*Statistically significant for p<0.05. Different letters in a row indicate the presence of statistically significant difference.

## DISCUSSION

In this research methodology, the authors have pursued a manner to handle images that, when the photos were digitally manipulated and so observed by the evaluators, these changes were as least perceptible as possible, giving to the observer a natural esthetic smile visual sensation. In the vast majority of researches in which digital images are manipulated, arguably the program of choice is Adobe Photoshop.^13^ ^,^ ^15^ ^-^ ^21^ Adobe Photoshop is an image editing program that has been marketed for many years because it has advanced features, but nevertheless often requires a qualified professional who know how to use this program for the digital images manipulation, to achieve a manipulated image as natural as possible. Due to this difficulty and so to the practicality that Keynote software offers, with simple resources that enables the own researcher to operate it and make the wanted digital images manipulation, in the present study Keynote was chosen, because the digital images manipulations showed as high quality as those performed with Adobe Photoshop and in some cases even better one. The maxillary incisors transfer and the ruler calibration have made the images of this study very similar to the patients’ teeth real size.

Canine is a tooth that has a prominent buccal bossa and the photograph can suffer some distortions in this area. Due to this it was decided not to make any digital manipulation because it would be closer to the real one and less perceptible to the evaluators than the digital manipulated photo. There was also a concern to standardize as much as possible the smile image attainment. The photos were taken only by a researcher at the same distance from the camera lens to the patients’ lips, under the same lighting conditions.

In the age comparison between the three groups (Table 1), laypersons were the youngest and general dentists and orthodontists had their ages statistically similar. Pithon et al^17^ showed that younger laypersons are more critical to the dental aesthetic than older ones, but even though they were the group with younger subjects, they were still less critical than dentists and orthodontists when evaluating smiles. When comparing the older and the younger groups (Table 4), the older groups gave lower scores than the younger one, confirming the fact that general dentists and orthodontists were the older subjects and laypersons were the youngest. A similar result was found in a study^22^ of the perception of smile attractiveness and its aesthetics standards variations, in which younger evaluators were more critical when judging smiles with diastema. Sriphadungporn and Chamnannidiadha^23^ also concluded that age impacts smile perception. However, to Kokich et al^13^ the professional evaluators’ years of experience and laypersons evaluators’ age did not influence the aesthetic perception.

In the comparison of smile changes among all groups and separately between the three groups (Table 2), laypersons had significant perception of 1-mm asymmetric changes in the maxillary right central incisor, but in other studies these asymmetric changes were noted from 2-mm asymmetry.^13^ ^,^ ^24^ This is similar to other study^25^ in which dental students were the evaluators and these asymmetries were noted from 2-mm changes. This findings suggests that any therapeutic attempt (orthodontic, aesthetic or surgical) to correct gingival margins asymmetries between 0.5 and 1,5mm may be an overreacted measure of dental professionals rather than an aesthetic appeal, since it seems not to be so relevant to laypersons.^24^

Some authors^13^ ^,^ ^26^ demonstrated that dental and gingival asymmetries have a negative impact on patient attractiveness. The results of the present study shows that gingival asymmetries are always less attractive than the symmetric changes, that is, when small changes occurs the evaluators show differences in their judgments, where symmetric changes are more difficult to identify. In another study, Kokich et al^16^ compared the dentists and laypersons perception to symmetrical dental changes, and noted that when the symmetrical gingival margin change was evaluated, none of the three groups (orthodontists, general dentists and laypersons) could distinguish between levels of gingival margin discrepancy. 

It was also noticed that maxillary central incisors always had a greater aesthetic relevance when compared to their corresponding lateral incisors, showing a greater visual impact to the laypersons, dentists and orthodontists’ eyes. Machado et al^19^ affirm that the maxillary central incisors are the key to a pleasant smile and its symmetry is of utmost importance for the smile aesthetics.

Based in these results, laypersons, general dentists and orthodontists had a greater perception of 1-mm changes in the gingival margin and when asymmetrical, these results are different to those found in other studies,^13^ ^,^ ^24^ in which laypersons perceived this difference in the gingival margin just from 2-mm changes. 

Therefore, if 1-mm changes in the gingival margins are perceptible and uncomfortable to the laypersons’ aesthetic perception, orthodontists must take a great care when finalizing their orthodontic cases because the patients’ esthetic demands are increasing.

It also has been observed that when comparing maxillary central incisors with their correspondent lateral incisors, always the same changes performed in central incisors were much more perceptible to evaluators than those performed on lateral incisors. Therefore, dentists and orthodontists must preserve and seek for the maximum maxillary central incisors aesthetics of their patients, because they are the most visible teeth in people’s eyes.

## CONCLUSIONS

Asymmetric gingival zeniths are less attractive than symmetrical gingival zeniths; gingival zeniths changes greater than 1mm are perceptible in the smile attractiveness, both by laypersons as general dentists and orthodontists. When comparing maxillary central incisors with maxillary lateral incisors, the aesthetic changes performed in the central incisors are more perceptible than those performed in lateral incisors, both symmetrical as asymmetrical. 

In a general way, orthodontists and general dentists are more critical in the evaluation and perception of gingival zenith changes, with the laypersons perceiving this change only from 1mm of maxillary right central incisor asymmetrical change. However, orthodontists’ perception is similar with the laypersons when the gingival zenith changes are performed symmetrically in the maxillary central incisors.
